# HTLV-1 Infection and Its Associated Diseases

**DOI:** 10.1155/2012/123637

**Published:** 2012-05-07

**Authors:** Mineki Saito, Pooja Jain, Kunihiro Tsukasaki, Charles R. M. Bangham

**Affiliations:** ^1^Department of Immunology, Graduate School of Medicine, University of the Ryukyus, 207 Uehara, Okinawa 903-0215, Japan; ^2^Department of Microbiology and Immunology, Drexel Institute for Biotechnology & Virology Research, Drexel University College of Medicine, 3805 Old Easton Road, Doylestown, PA 18902, USA; ^3^Department of Hematology, Atomic Bomb Disease Institute, Graduate School of Biomedical Science, Nagasaki University, 1-12-4 Sakamoto, Nagasaki 852-8523, Japan; ^4^Department of Immunology, Wright-Fleming Institute, Imperial College London, London W2 1PG, UK

It has been three decades since the discovery of human T-cell leukemia virus type 1 (HTLV-1), which is the first human retrovirus etiologically associated with an aggressive malignancy of CD4+ T cells known as adult T-cell leukemia (ATL) and a disabling chronic inflammatory disease of the central nervous system known as HTLV-associated myelopathy/tropical spastic paraparesis (HAM/TSP). HTLV-1 infection is of particular interest to the field of immunology as well as virology, because HTLV-1 is never eliminated from the host in spite of a vigorous cellular and humoral immune responses against the virus, and it causes no disease in the majority (around 95%) of the infected subjects (asymptomatic carriers: ACs). Although accumulating evidence suggests the importance of complex virus-host interactions and the host immune response in determining the risk and timing of disease, the precise mechanism of disease pathophysiology is incompletely understood and the treatment is still unsatisfactory. The patients with ATL have a poor prognosis and approximately 40 percent of HAM/TSP patients become wheelchair-bound during their clinical course. In this special issue on “HTLV-1 Infection and Its Associated Diseases,” we have invited nine papers that address the recent developments in HTLV-1 research in order to elucidate the pathogenetic mechanisms and to identify effective means of treatment and prevention of HTLV-1 associated diseases. 

Three papers describe the important advances in understanding the molecular and virological aspects of HTLV-1. One paper explains the significant role of HTLV-1 basic leucine zipper factor (HBZ), a regulatory protein encoded in the minus strand of the HTLV-1 genome, in the viral pathogenesis. Another paper summarizes the comparative biology of HTLV-1 and HTLV-2. The paper by N. Aliya et al. discusses the possible interplay between HTLV-1 infection and miRNA pathways. Two papers describe the clinical trials and treatment for ATL from different groups, to give contrasting views on the current state of knowledge and current approaches to this subject. There is a paper that provides an overview of recent developments in HAM/TSP research, while another paper summarizes the current understanding of the host immune response against HTLV-1 infection, especially the potential importance of HTLV-1-specific CTLs in protection against ATL. Two clinical studies for ATL are also included. One of them describes the clinical and pathological characteristics of seven patients with HTLV-1-positive large B-Cell lymphoma, while another one discusses the hypercalcemia that is frequently observed in ATL patients. 

The papers in this special issue demonstrate important recent progress in HTLV-1 research and will bring the reader up to date with the current understanding of HTLV-1 infection and its associated diseases.

**Mineki Saito** **Mineki Saito** **Pooja Jain** **Pooja Jain** **Kunihiro Tsukasaki** **Kunihiro Tsukasaki** **Charles R. M. Bangham** **Charles R. M. Bangham**

